# Feasibility of postprandial optical scattering of lipoproteins in blood as an optical marker of cardiovascular disease risk: modeling and experimental validation

**DOI:** 10.1117/1.JBO.28.6.065002

**Published:** 2023-06-08

**Authors:** Anahita Pilvar, Declan W. Smith, Jorge Plutzky, Darren Roblyer

**Affiliations:** a Boston University, Department of Electrical and Computer Engineering, Boston, Massachusetts, United States; b Boston University, Department of Physics, Boston, Massachusetts, United States; c Brigham and Women’s Hospital and Harvard Medical School, Department of Medicine, Boston, Massachusetts, United States; d Boston University, Department of Biomedical Engineering, Boston, Massachusetts, United States

**Keywords:** optical scattering, blood optical properties, diffuse optics, blood lipids, lipoproteins, cardiovascular disease, spatial frequency domain imaging

## Abstract

**Significance:**

Blood lipid levels (i.e., triglycerides (TGs) and cholesterol) are a strong predictor of cardiovascular disease (CVD) risk. Current methods for measuring blood lipids require invasive blood draws and traditional lab testing, limiting their practicality for frequent monitoring. Optical measurements of lipoproteins, which carry TG and cholesterol in blood, may lead to simpler invasive or non-invasive methods for more frequent and rapid blood lipid measurements.

**Aim:**

To investigate the effect of lipoproteins on optical properties of blood before and after a high-fat meal (i.e., the pre- and post-prandial state).

**Approach:**

Simulations were performed using Mie theory to estimate lipoprotein scattering properties. A literature review was conducted to identify key simulation parameters including lipoprotein size distributions and number density. Experimental validation of *ex-vivo* blood samples was conducted using spatial frequency domain imaging.

**Results:**

Our results indicated that lipoproteins in blood, particularly very low-density lipoproteins and chylomicrons, are highly scattering in the visible and near-infrared wavelength region. Estimates of the increase in the reduced scattering coefficient ($\mu_{s}^{\prime }$) of blood at 730 nm after a high-fat meal ranged from 4% for a healthy individual, to 15% for those with type 2 diabetes, to up to 64% for those suffering from hypertriglyceridemia. A reduction in blood scattering anisotropy (g) also occurred as a function of TG concentration increase.

**Conclusion:**

These findings lay the foundation for future research in the development of optical methods for invasive and non-invasive optical measure of blood lipoproteins, which could improve early detection and management of CVD risk.

## 1 Introduction

Plasma lipoproteins, which are complex biochemical particles that transport hydrophobic lipids through the blood, are largely unrecognized as a potential optical biomarker for the evaluation of cardiovascular health. Lipoprotein particles are known to be partially responsible for blood plasma turbidity, and they very likely affect blood optical properties. Plasma levels of triglyceride (TG) and cholesterol, which are packaged and transported in blood within lipoproteins, are strongly predictive of future cardiovascular events, including heart attack and stroke.^1^^,^^2^ Currently, measurement of these plasma blood lipids requires invasive blood draw and traditional laboratory-based testing. Label-free optical measurements of blood lipoproteins may have important implications for cardiovascular health assessment, especially for those suffering from dyslipidemias, such as type 2 diabetes.^3^

The optical properties of blood have been a topic of interest for many years with implications in variety of diagnostic and therapeutic applications and blood quality assessment. Both the absorption and scattering properties of blood are dominated by red blood cells (RBCs), which account for ∼36% to 54% of human blood.^4^ Hemoglobin, which accounts for ∼95% of the volume of RBCs, is responsible for the optical absorption of blood at visible and near infrared (NIR) wavelengths.^5^ At longer wavelengths (>1000  nm), blood absorption is dominated by the optical absorption of water, which accounts for around 90% of blood plasma. The scattering properties of blood are largely a consequence of the refractive index mismatch between RBCs and blood plasma, but are also affected by the size, shape, orientation, and concentration of the RBCs as well as other cells, proteins, vesicles, and other scattering particles. There have also been investigations of how glucose can alter blood and tissue optical scattering, with prior work demonstrating a decrease in tissue optical scattering due to elevated levels of glucose caused by a closer matching of refractive index between the extracellular fluid and cellular membrane.^6^

While less explored, lipoproteins are also likely to alter blood optical properties, especially optical scattering. The TG-rich lipoproteins (TRL), including chylomicrons and very low-density lipoproteins (VLDL), are large (20 to 1200 nm in diameter)^7^^,^^8^ and have a substantial index of refraction mismatch from plasma. Due to their size and refractive index, TRLs provide a strong source of Mie scattering to visible and NIR light. There has been very little prior work investigating how naturally occurring lipoproteins affect blood optical properties, representing a potential opportunity for new invasive and non-invasive optical health monitoring techniques that could exploit this contrast.

In this work, we used computational modeling and experimental measurements to quantify the effect of lipoproteins on the optical properties of blood. In the sections below we build towards a realistic estimate of the changes in human blood optical properties after a high-fat meal. We first describe the basic properties of plasma lipoproteins. We then describe a series of simplified experiments to measure the effects of added lipoprotein-like particles on the optical properties of blood using a technique called spatial frequency domain imaging (SFDI).^9^ We then show how Mie theory was used to validate the experimental results and describe our strategy to account for the effect of so-called “dependent scattering” from the close spacing of RBCs in plasma. Next, we report on an extensive literature review used to estimate expected fasting and postprandial (i.e., after a meal) lipoprotein size distributions, concentrations, and temporal dynamics. Finally, we show how these parameters were used as part of a more comprehensive model to provide predictions of expected scattering changes in blood after a high-fat meal for both healthy individuals and patients with dyslipidemias. Together, this work provides a framework for the development of optical methods to detect lipoproteins in blood.

## 2 Basic Properties of Plasma Lipoproteins and Their Synthesis

Plasma lipoproteins are classified according to their density into several particle types, including chylomicrons, VLDL, low density (LDL), intermediate-density (IDL), and high-density (HDL) lipoproteins. Of these, chylomicrons have the lowest density and the largest size, typically ranging between 75 and 1200 nm.^7^^,^^8^ After fat intake, dietary lipids including TG and cholesterol are packaged into large TG rich lipoproteins (TRL) (i.e., chylomicrons) in enterocyte cells in the small intestine and enter the bloodstream via the lymphatic ducts.^7^ Chylomicrons deliver dietary fat to tissues throughout the body, after which they are transformed into smaller particles called chylomicron remnants, which are taken up by the liver. VLDLs are also TRLs, and have lower TG and higher cholesterol levels compared to chylomicrons. VLDLs are the endogenous source of TG and are synthesized in the liver before entering the blood circulation. They are smaller than chylomicrons with diameters ranging from 30 to 80 nm.^7^ Both VLDL and Chylomicron are spherical micelles composed of a monolayer phospholipid shell that protects the hydrophobic core of TG and cholesterol.

**Fig. 1:**
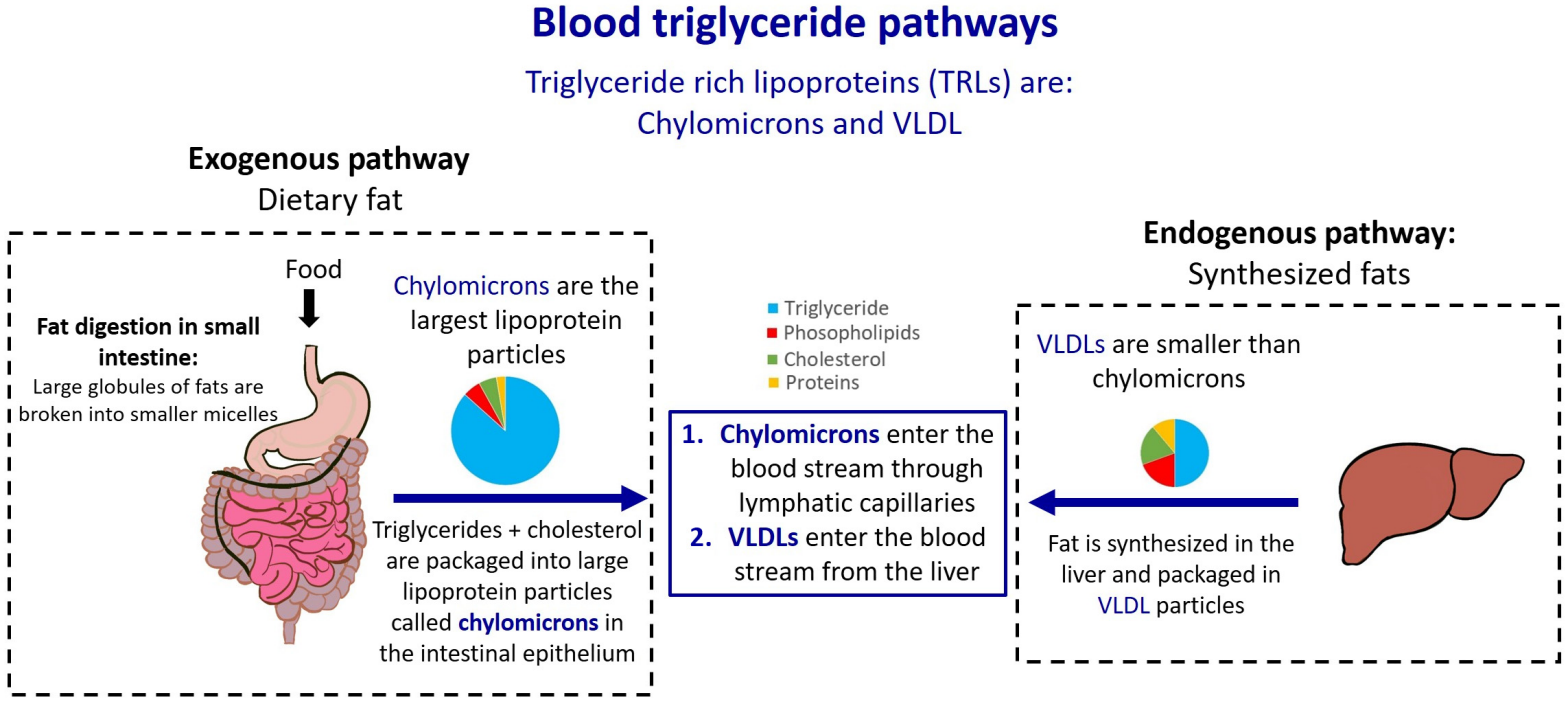
Blood TG pathways: chylomicron and VLDL are the main source of blood TGs. Chylomicrons enter the blood through the small intestine after dietary fat intake and hence they are the exogenous source of blood TG. VLDLs enter the blood through the liver and they are the endogenous source of blood TG.

Figure 1 summarizes the blood TG pathways including the exogenous pathway, in which ingested fats are packages in chylomicrons, as well as the endogenous pathway, in which the liver produces VLDLs. Chylomicron and VLDL particles account for most of the blood TG levels and their concentrations are highly influenced by food intake. As we will show, these TRL particles are a strong source of Mie scattering due to their size and other important properties. Smaller cholesterol-rich particles, such as LDL, IDL, and HDL are unlikely to provide substantial optical scattering in the visible and NIR wavelength bands.

## 3 Experimental Measurements of Lipoproteins-like Particles in Blood

To assess the effect of lipoprotein-like particles on blood optical properties, an *ex-vivo* experiment was conducted using fresh bovine blood (Carolina Biological Supply) and intralipid (INTRALIPID^®^ 20%) as surrogate for TRLs. Intralipid is a fat emulsion composed of soybean oil, egg yolk phospholipids, glycerin, and water and is used as an intravenous source of calories and essential fatty acids for several therapeutic and nutrition indications, such as nutrition supply for patients with essential fatty acid deficiency. Intralipid is available at different lipid concentrations, described by the percentage of soybean oil (mass) in the volume of emulsion. For example, 1000 ml of intralipid 20% contains 200 g of soybean oil, which is >70% TG.^10^ The size distribution of intralipid particles has been previously quantified through electron microscopy, with reported intralipid particle sizes ranging from 25 to 675 nm with an exponentially decreasing size distribution and an average particle size of 97 nm.^11^ The refractive index (RI) of intralipid micelles is ∼1.46, which is similar to the RI reported for chylomicron.^11^^,^^12^ Due to its particle size range and composition, intralipid closely resembles blood chylomicrons.

SFDI was used to measure the optical properties of blood and the effect of lipids on blood absorption and scattering experimentally. SFDI is a non-contact diffuse optical modality, which allows the extraction of blood optical properties from a whole blood sample without a need of centrifugation. In SFDI, 1-D sinusoidal patterns of light at different wavelengths and spatial frequencies are projected on the sample and the reemitted light is captured by a camera. The captured images are processed resulting in maps of optical properties [absorption ($\mu_{a}$) and reduced scattering coefficient ($\mu_{s}^{\prime }$)] at each measurement wavelength.^13^ We previously fabricated a dual-LED-based NIR-SWIR SFDI system.^9^ For this study we added a third NIR wavelength to the system. Our current version of the SFDI system utilizes three LEDs at 730, 880, and 1100 nm as the illumination source. A digital micromirror device (LC4500, Keynote Photonics) was used to generate the spatial patterns of light. An InGaAs camera (Triwave, Infrared Laboratories, Inc., Peabody, Massachusetts, United States) with a wide range of optical sensitivity was used as the detector.

The concentration of intralipid was titrated in bovine blood from 0.1% to 1% in increments of 0.1%. These intralipid concentrations corresponds to TG levels in blood ranging from a healthy fasting state to a very high state representative of a patient suffering from hypertriglyceridemia, a condition in which lipoproteins are not rapidly absorbed into tissue from the blood.^14^ The optical properties of pure blood and blood-intralipid mixture were measured at each intralipid concentration using our SFDI system. In each case, a 12-ml sample was prepared in a cubic well in an optically diffuse phantom as in our prior work.^15^

**Fig. 2:**
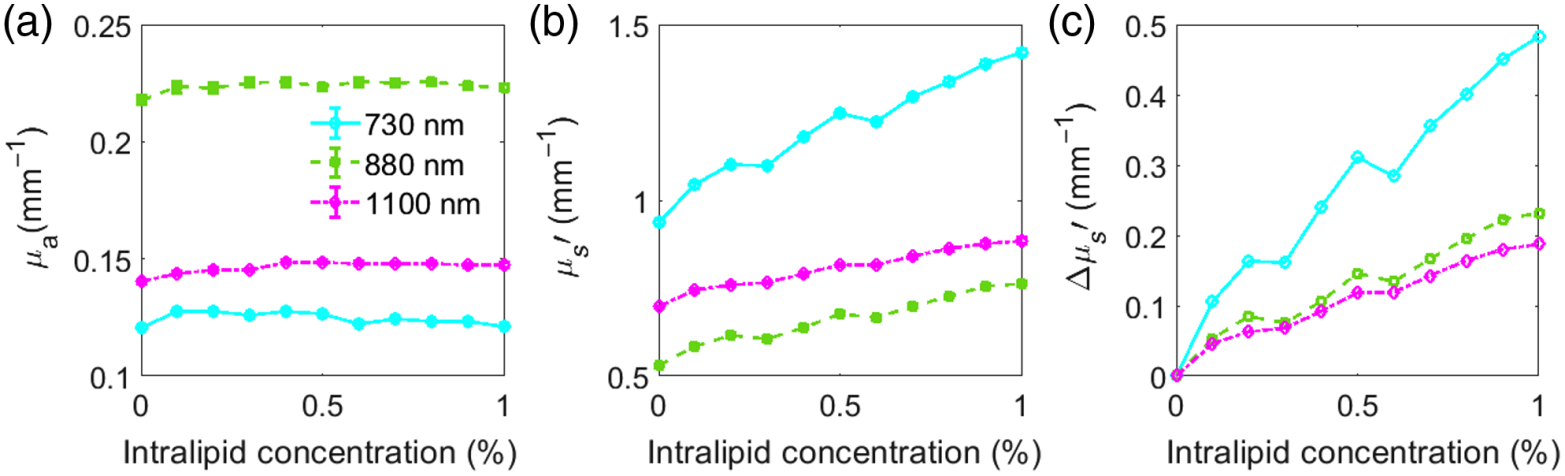
Intralipid in bovine blood titration experiment results. (a) Measured absorption coefficient at three wavelengths showing no appreciable change as intralipid concentration increases from 0.1% to 1%. (b) Reduced scattering coefficient increases with intralipid concentration for all measured wavelengths. (c) $\Delta \mu_{s}^{\prime }$ shows larger increase for shorter NIR wavelengths.

The results, as shown in Fig. 2(a), indicate that the absorption properties of blood are largely unchanged by the addition of intralipid at these concentrations. This observation is supported by the estimated changes predicted using Beer’s law and known extinction coefficients, which predict a less than $10^{-4}\;{\mathrm{mm}}^{-1}$ increase in $\mu_{a}$ for a 0.1% increase in lipid concentration at these wavelengths. In contrast, the reduced scattering coefficient of blood increased with increasing intralipid concentration [Fig. 2(b)], with larger $\Delta \mu_{s}^{\prime }$ observed at shorter, NIR wavelengths compared to the longer, SWIR wavelengths [Fig. 2(c)]. We observed a $\mu_{s}^{\prime }$ increase of $0.089\;{\mathrm{mm}}^{-1}$ (9.5%), $0.042\;{\mathrm{mm}}^{-1}$ (8%) and $0.036\;{\mathrm{mm}}^{-1}$ (5.2%) corresponding to a 0.1% increase in intralipid concentration at 730, 880, and 1100 nm, respectively. It should be noted that the $\mu_{s}^{\prime }$ spectra do not follow power law behavior for the ex-vivo blood measurements due to the predominant effect of large RBCs on optical scattering, resulting in scattering oscillation at these wavelengths when the measurement wavelength is small compared to the size of the scattering particles.

**Fig. 3:**
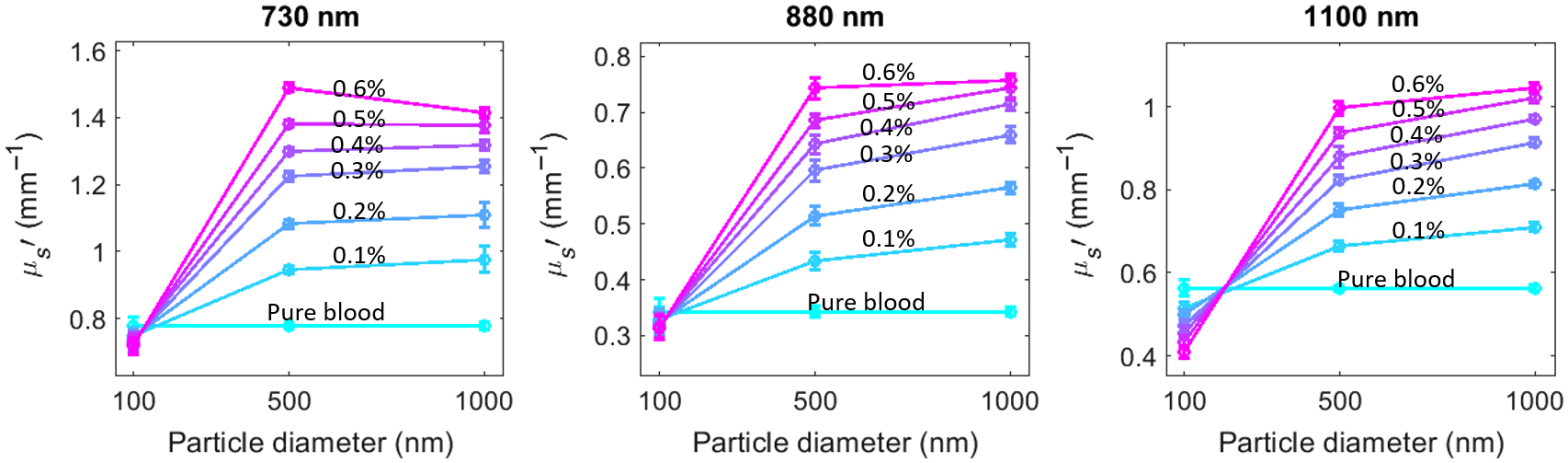
Microsphere bead in blood titration experiment at 730, 880, and 1100 nm. The results show the effect of size of the added particles on blood $\mu_{s}^{\prime }$ at different concentrations. The largest $\mu_{s}^{\prime }$ increases occur from 100 to 500 nm. Bead concentration is indicated as volume percentage of bead in blood.

Since the size of chylomicron particles is also affected by a meal,^16^^,^^17^ we next investigated the effect of particle size on blood optical scattering through a mono-disperse polystyrene microsphere titration experiment. In this study, microsphere beads of three sizes were used: 100, 500, and 1000 nm, mimicking small, medium, and large lipoproteins. We note that the refractive index of microspheres is ∼1.57 at the measured wavelengths which is higher than the refractive index of lipoproteins and intralipid micelles, which range between ∼1.46 and 1.5.^12^^,^^18^ SFDI measurements were taken of the mixture of microsphere particles in bovine blood with bead concentration (V/V) ranging from 0.1% to 0.6% with 0.1% increments. Figure 3 illustrates the effect of particle concentration and size on the $\mu_{s}^{\prime }$ of blood, showing that increasing the size of the added particles leads to an increase in blood $\mu_{s}^{\prime }$, with a more pronounced effect when the bead size increases from 100 to 500 nm.

## 4 Estimation of Blood $\mu_{s}^{\prime }$ Alterations with Mie Theory

Mie theory was used to model the experimental results above. Mie theory describes the scattering of electromagnetic plate wave by homogenous spherical particles. The following equation can be used to approximate the reduced scattering coefficient of multisized particles (shown with index p in the summation) using the Mie theory framework:^19^

$$\mu_{s}^{\prime }(\lambda )=N_{0}\overset{p}{\underset{i=1}{\sum }}f(a_{i})(\pi a_{i}^{2})Q_{\mathrm{scat}}(m,a_{i},\lambda )[1-g(m,a_{i},\lambda )],$$ (1)

$$m=\frac{n_{1}}{n_{0}}.$$ (2)

Here, $N_{0}$ refers to the number density of the scattering particles, $f(a_{i})$ refers to the normalized particle size distribution, a is the particle radius, $Q_{\mathrm{scat}}$ is the scattering efficiency derived from Mie theory, λ is the wavelength, and m is the relative refractive index where $n_{1}$ and $n_{0}$ are the refractive indices of the particles and the surrounding medium, respectively. The g parameter is the anisotropy factor which can also be predicted with Mie theory. The summation is used to add the contribution of all particles with size $a_{i}$ after adjusting the results by their distribution factor ($f(a_{i})$).

The use of Mie theory to predict blood optical properties comes with a caveat of dependent scattering caused by RBCs. Dependent scattering occurs in environments where the distance between scattering particles is relatively small compared to the size of the particles.^20^ Mie theory is only applicable when the particle spacing is at least three to five times larger than the particle diameters, a criterion that is not satisfied in blood where RBCs are the dominant source of scattering.^21^ Previous studies have applied correction factors to account for the dependent scattering effect of RBCs. One such correction factor is the Twersky factor, which involves multiplying a constant factor dependent on the shape and concentration of the particles to the Mie simulation results^22^

$$W(m)=\frac{(1-h{)}^{m+1}}{{\left(1+h(m-1)\right)}^{m-1}}.$$ (3)

In the Twersky equation for blood, h refers to the blood hematocrit and m is the packing factor that relates to the shape of the RBCs. In its simplest form, m is defined as an integer value of 1, 2, or 3 for particles that are plate-like, cylindrical, or spherical, respectively. Some studies, such as the study on intralipid dependent scattering at high concentrations by Aernouts et al. used non-integer values for m which were empirically determined by fitting the results of the simulations to the experimental results.^23^

Here, Mie theory was used to predict the $\mu_{s}^{\prime }$ at each lipid concentration in the bovine blood-intralipid titration experiment. The size distribution of intralipid particles reported by Van Staveren et al was used for all Mie simulations.^11^ A refractive index of 1.46 was assigned to the intralipid particles and 1.345 to the media.^11^^,^^24^

Mie simulations were only conducted for intralipid particles. The refractive index of blood was used as the $n_{0}$ in Eq. (1). To correct for dependent scattering, the Mie predicted $\mu_{s}^{\prime }$ at each intralipid concentration was multiplied by the Twersky factor. The results were then added to the $\mu_{s}^{\prime }$ of the pure bovine blood measured with SFDI to mimic intralipid in bovine blood experiment at each concentration. The hematocrit level of the bovine blood was measured using an auto blood analyzer (HEMAVET 950FS, Drew Scientific, Florida, United States) and found to be 0.33. The packing dimension of the Twerksy factor was found empirically by fitting the results of the Mie theory multiplied by the Twersky factor for various concentrations to the measured $\mu_{s}^{\prime }$ at each measurement wavelength. Packing factors of 0.97, 1.78, and 1.61 were found to most effectively match the Mie simulations to the experiment results at 730, 880, and 1100 nm, respectively.

**Fig. 4:**
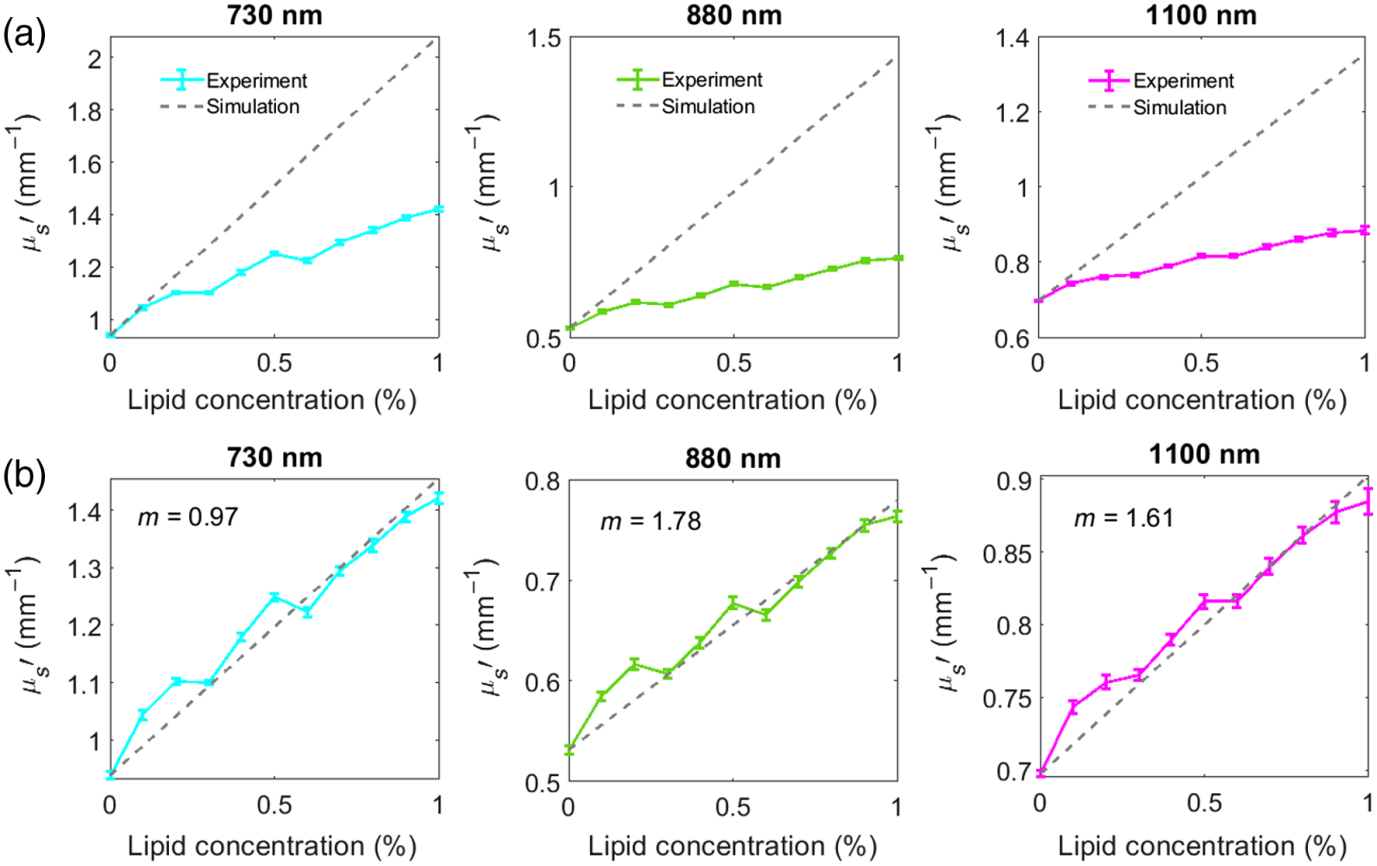
(a) $\mu_{s}^{\prime }$ results from titration experiments are shown in solid lines and Mie simulation results from the same lipid concentrations added to the baseline pure blood $\mu_{s}^{\prime }$ are shown in dashed lines at 730, 880, and 1100 nm. The experiment results do not match the simulations due to the dependent scattering caused by RBCs. (b) Mie predicted $\mu_{s}^{\prime }$ matches the experiment results after correction by Twersky factor to account for dependent scattering.

Figure 4(a) shows the results of the experiment and the simulations for each of the three wavelengths. The results indicate that the experiment results do not match the Mie simulation before applying the Twerksy factor, with the discrepancy between the experiment and the simulation increasing as the lipid concentration increases. Figure 4(b) shows the experiment and simulation results after applying the Twerksy factor to the output of the Mie equation. These results suggest that Mie theory combined with a dependent scattering correction factor can predict the $\mu_{s}^{\prime }$ for a given concentration of scattering particles in blood. The mean absolute error between the experiment and the simulation results reduced from $0.30\;{\mathrm{mm}}^{-1}$, $0.32\;{\mathrm{mm}}^{-1}$, and $0.22\;{\mathrm{mm}}^{-1}$ to $0.027\;{\mathrm{mm}}^{-1}$, $0.012\;{\mathrm{mm}}^{-1}$, and $0.098\;{\mathrm{mm}}^{-1}$ after we applied the Twersky factor to the simulated data at 730, 880, and 1100 nm, respectively.

**Fig. 5:**
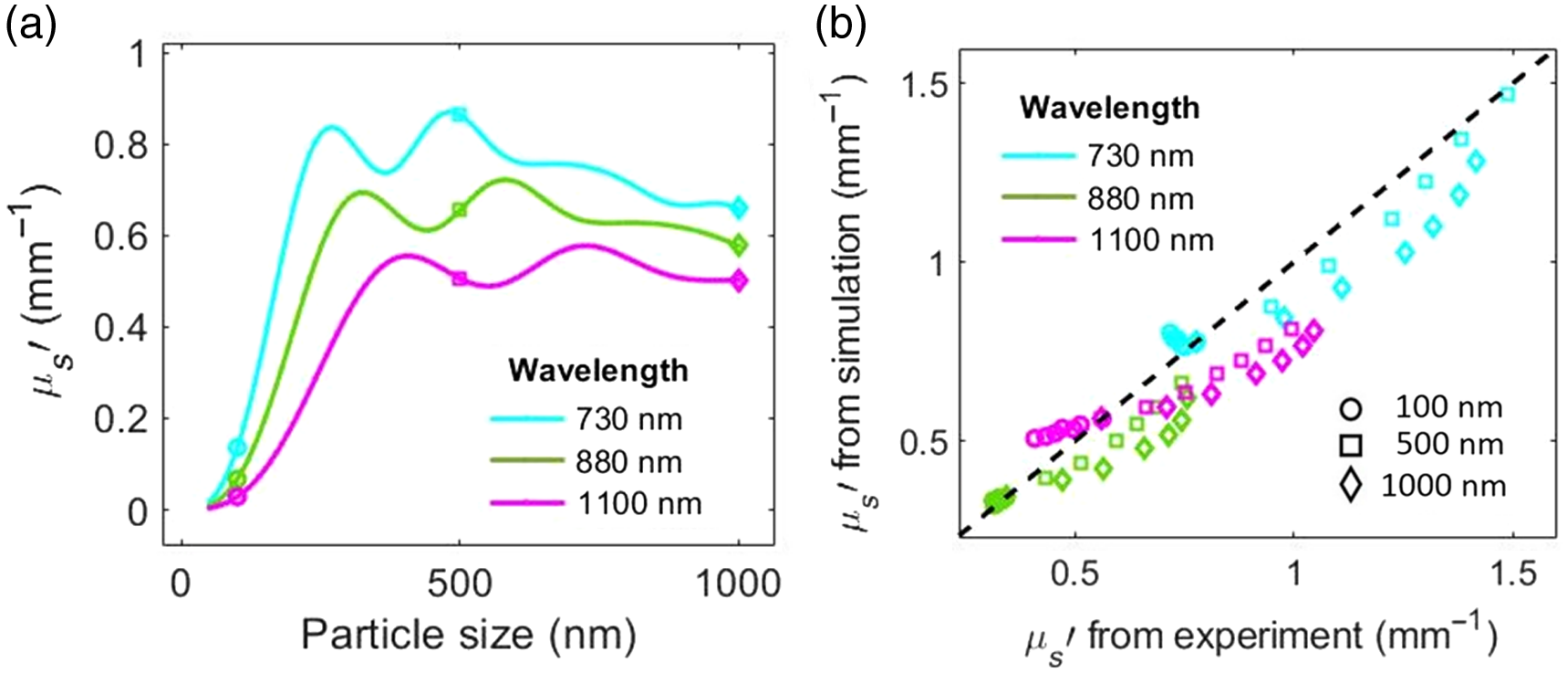
(a) Mie simulation results show the effect of particle size on $\mu_{s}^{\prime }$ at three simulated wavelengths. The markers show the particle sizes that were used in the bead experiment. (b) Comparison between the measured $\mu_{s}^{\prime }$ and the simulated $\mu_{s}^{\prime }$ at different bead size, concentration, and wavelengths. Different colors correspond to different wavelengths and different marker shapes correspond to different bead sizes. Each data point corresponds to a different concentration from 0.1% to 0.6% (V/V) of bead in bovine blood. The black dashed line is the identity line.

The effect of particle size on $\mu_{s}^{\prime }$ was tested by conducting Mie simulation for mono-disperse particles with diameter of 50 to 1000 nm at one selected concentration (0.3% V/V). Figure 5(a) demonstrates the simulated effect of particle size on $\mu_{s}^{\prime }$ at the three wavelengths. The simulation results supports our observation from the beads-in-bovine-blood titration experiment (Fig. 3), where larger particle size results in an increase in the measured $\mu_{s}^{\prime }$. The effect of size on $\mu_{s}^{\prime }$ is more significant for smaller particle sizes relative to the wavelength. The simulated $\mu_{s}^{\prime }$ for particles with similar refractive index as chylomicrons (∼1.46) shows a similar trend with size but with lower absolute values. We then compared the simulated $\mu_{s}^{\prime }$ to the measured $\mu_{s}^{\prime }$ from Sec. 3 at different size, concentration, and wavelengths used in the experiment. Figure 5(b) shows that the simulated $\mu_{s}^{\prime }$ closely follows the experimental $\mu_{s}^{\prime }$ measured with SFDI. The intraclass correlation coefficient [ICC(1-1)] shows a strong agreement between the experimental and simulated data (ICC=0.92, p-value<0.0001).

## 5 Chylomicron and VLDL Scattering Parameters from Literature

The sections above provide an initial evaluation of how lipoprotein-like particles affect blood optical properties. To gain a better understanding of how human lipoproteins affect optical scattering of blood in the postprandial state, it is necessary to have knowledge of the size, number density, and refractive index of the TRLs in the blood pre- and post-meal. Prior research has focused on blood TG content when studying blood lipids, and less is known about chylomicron concentration and the distribution of chylomicrons and VLDLs. Here, we review the existing literature on the TRLs and use this information to generate a size distribution with accurate particle concentration for these lipoproteins. The information on size and concentration as well as the refractive index of TRLs were used to predict the impact of TRL alterations on blood optical scattering pre- and post-meal using Mie theory.

Table 1 summarizes the reported literature properties for TRLs and blood TG content in the fasting state. The concentration of chylomicron and VLDL particles is directly related to the blood TG level, as most of the blood TG is carried by these particles. If a size distribution is assumed for chylomicron and VLDL particles, the blood TG level can be converted into the concentration of TRL particles of each size. It was assumed that chylomicrons and VLDLs have an exponentially decreasing size distribution,^11^^,^^12^ with chylomicrons spanning between 75 and 1200 nm and VLDL particles spanning 30 to 80 nm.^7^^,^^8^ An exponential size distribution was generated for each particle with an average size of 100 nm for chylomicrons and 50 nm for VLDLs

$$f(a_{i})=\frac{\exp(-\frac{a_{i}}{a})}{a},$$ (4)

where $f(a_{i})$ is the distribution function with respect to particle size and a is the size parameter related to the average size of the distribution.

**Table 1:** Reported literature properties for TRLs and TG in the fasting state as well as model parameters.

Property	Reported values	Reference	Notes	Model parameters
Particle diameter	Chylomicron: 75 to 1200 nm	Gotto et al., methods in enzymology, 1986^7^		Chylomicron average size: 100-nm
	VLDL: 30 to 80 mm	Rensen et al., advanced drug delivery reviews, 2001^8^		VLDL average size: 50 nm
Particle size distribution	Exponential	Van Staveren et al., Appl. Opt. 1991^11^	Assume chylomicrons have the same size distribution shape to intralipid	Exponential
		Chernova et al., J Biophotonics, 2018^12^		
Density (g/ml)	Chylomicron: <0.96	Gotto et al., methods in enzymology, 1986^7^^a^	a. Reported 0.93 for chylomicron	Chylomicron: 0.93
	VLDL: 0.96 to 1.006	Rensen et al., advanced drug delivery reviews, 2001^8^		VLDL: 1
TG content (%w/w)	Chylomicron: 80% to 95%	Rensen et al., advanced drug delivery reviews, 2001^8^		Chylomicron: 90%
	VLDL: 45% to 65%			VLDL: 50%
Refractive index	Chylomicron: 1.46^b^	Chernova et al., J Biophotonics. 2018^12^	b. No information on VLDL is available	RI: 1.46 for both VLDLs and chylomicrons
Plasma fasting TG (mg/dL)	Normal: <150 mg/dL	2001 NCEP guidelines^25^		Healthy: 50 mg/dL
	Borderline high: 150 to 199 mg/dL			Diabetes: 200 mg/dL
	High: 200 to 499 mg/dL			Hypertriglyceridemia: 200 mg/dL
	Very high: >500 mg/dL			

To calculate the concentration of the particles, it was first assumed that the blood TG content is distributed between chylomicron and VLDL particles, with 30% of fasting TG coming from chylomicrons and 70% from VLDL.^26^ Next, chylomicron and VLDL mass concentrations were calculated for the corresponding blood TG concentrations, assuming that 90% of chylomicrons and 50% of VLDLs mass are TG.^8^ The volume concentration of TRLs was then calculated by dividing the mass concentration of the particles by their volume density. The volume density of chylomicrons and VLDLs are approximately 930 and 1000  mg/ml, respectively.^8^ Using the generated size distribution for these two particles, we then calculated the total number of each particle at each size by dividing the volume concentration of chylomicrons and VLDLs by the total volume of these particles from the normalized size distribution

$$N=\frac{P}{\sum_{i}f(a_{i})V(a_{i})},$$ (5)

where N refers to the total number of particles (CM or VLDL), P is the total volume of the particles, f is the normalized size distribution, V is the volume of the particles with size $a_{i}$, and i is the number of particles in the size distribution.

**Fig. 6:**
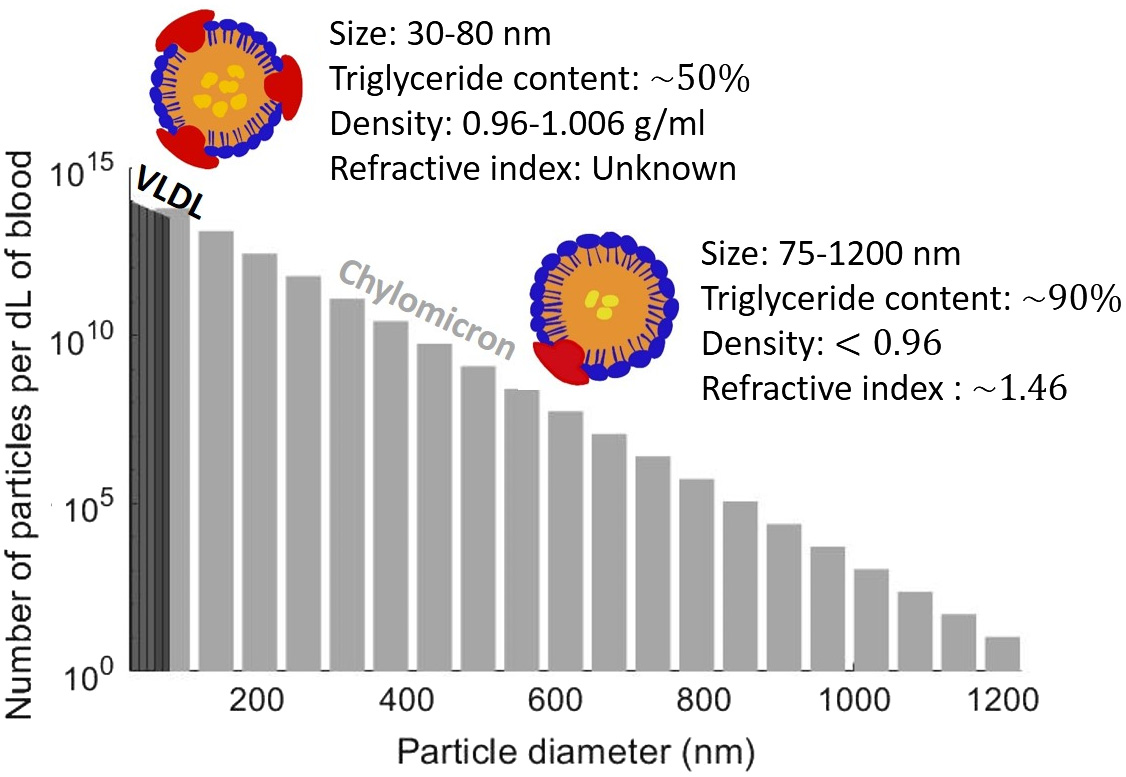
VLDL and chylomicron size distribution for 100  mg/dL of blood TG.

We then generated a distribution of chylomicron and VLDL particles for any concentration of fasting TG in blood by multiplying the total number of each particle by the normalized size distribution $f(a_{i})$. Figure 6 shows this distribution of VLDLs and chylomicrons for blood TG level of 100  mg/dL.

Table 2 summarizes the changes in TG content and TRL properties that may occur after a meal based on literature. An increase in blood TG levels primarily results in changes in chylomicron distribution, resulting in larger average particle size, higher refractive index, and higher number density.^12^^,^^16^^,^^17^

**Table 2:** Reported literature properties for TRLs and TG in the postprandial state.

Property	Reported values	Reference	Notes	Model parameter
Blood TG	Healthy: <175 mg/dL	Nordestgaard et al., *European Heart Journal*, 2016^27^		Healthy: 100 mg/dL
	High: 175 to 880 mg/dL			Diabetes: 400 mg/dL
	Hypertriglyceridemia > 880 mg/dL			Hypertriglyceridemia: 1200 mg/dL
Peak refractive index	Chylomicron: ∼1.5	Chernova DN et al., *J Biophotonics*, 2018^12^	No information on RI of VLDLs is available	CM RI: 1.5
				VLDL RI: 1.46 (no change)
	VLDL: —			
Peak average size	Chylomicron: ∼200 nm	Milan et al., *British Journal of Nutrition*, 2016^17^	Effect of meal on VLDL particle size is minimal	Chylomicron average size: 200 nm
	VLDL: ∼60 nm	Wojczynski et al., *Lipids in Health and Diseases*, 2011^16^		VLDL average size: 50 nm (no change)

A TG level of <150  mg/dL is considered to be normal in the fasting state.^25^ For a healthy individual, the TG levels in the blood increases and reaches its peak 2 to 4 hrs after a meal. TG content can rise up to 175  mg/dL, depending on the meal and the person’s response to the fat intake.^27^ However, for individuals with type 2 diabetes or hypertriglyceridemia, both the baseline and postprandial TG levels can be much higher. A baseline TG level of 200  mg/dL is considered high, and levels above 500  mg/dL are considered very high. In the postprandial state, TG levels may reach as high as 1000  mg/dL or more for subjects with such conditions.^27^

In addition to concentration, the size of the chylomicrons also increases after a meal. One study showed that the size increase ranges from 90 to 120 nm, with younger subjects showing larger increases compared to older subjects.^17^ Additionally, the size of the smaller TRLs, VLDLs, increase by about 10 nm after a meal.^16^ The increase in particle size is related to the amount of TG carried by the particles, with larger particles carrying more TGs.

The refractive index of the TRLs has also been shown to change with alterations in their size and composition. Particles with higher TG content have higher refractive index compared to the particles with lower TG content. The mean refractive index of chylomicrons in the fasting state is reported to be 1.46, and increases to around 1.5 after a meal.^12^ However, there is limited information on the refractive index of VLDLs and how it changes after a meal in the prior literature.

## 6 Estimation of Blood $\mu_{s}^{\prime }$ Changes After a Meal

Having the above information on TRL size distribution, concentration, and refractive index allows for the estimation of expected blood $\mu_{s}^{\prime }$ during fasting and after a high fat meal. We first simulated the whole blood environment by assuming a single particle size for RBCs. The non-spherical shapes of RBCs does not satisfy the spherical particle assumption in Mie theory, however, previous studies have attempted to overcome this limitation by assuming a spherical shape for RBCs with a diameter that corresponds to their equivalent volume.^28^ For these simulations a diameter of 5.56  μm and a number density of $0.005\text{ cell}/\mu m^{3}$ (correspond to 0.45 hematocrit) was assumed for RBCs.^29^ The refractive index for RBCs was assumed to be 1.4.^29^ Using the distribution of chylomicrons, VLDLs, and RBCs, the effect of postprandial lipid alterations on blood scattering properties was estimated using Mie theory combined with the Twerksy correction factor for dependent scattering. To calculate the Twersky factor, the packing factors determined in Sec. 4 were used.

**Fig. 7:**
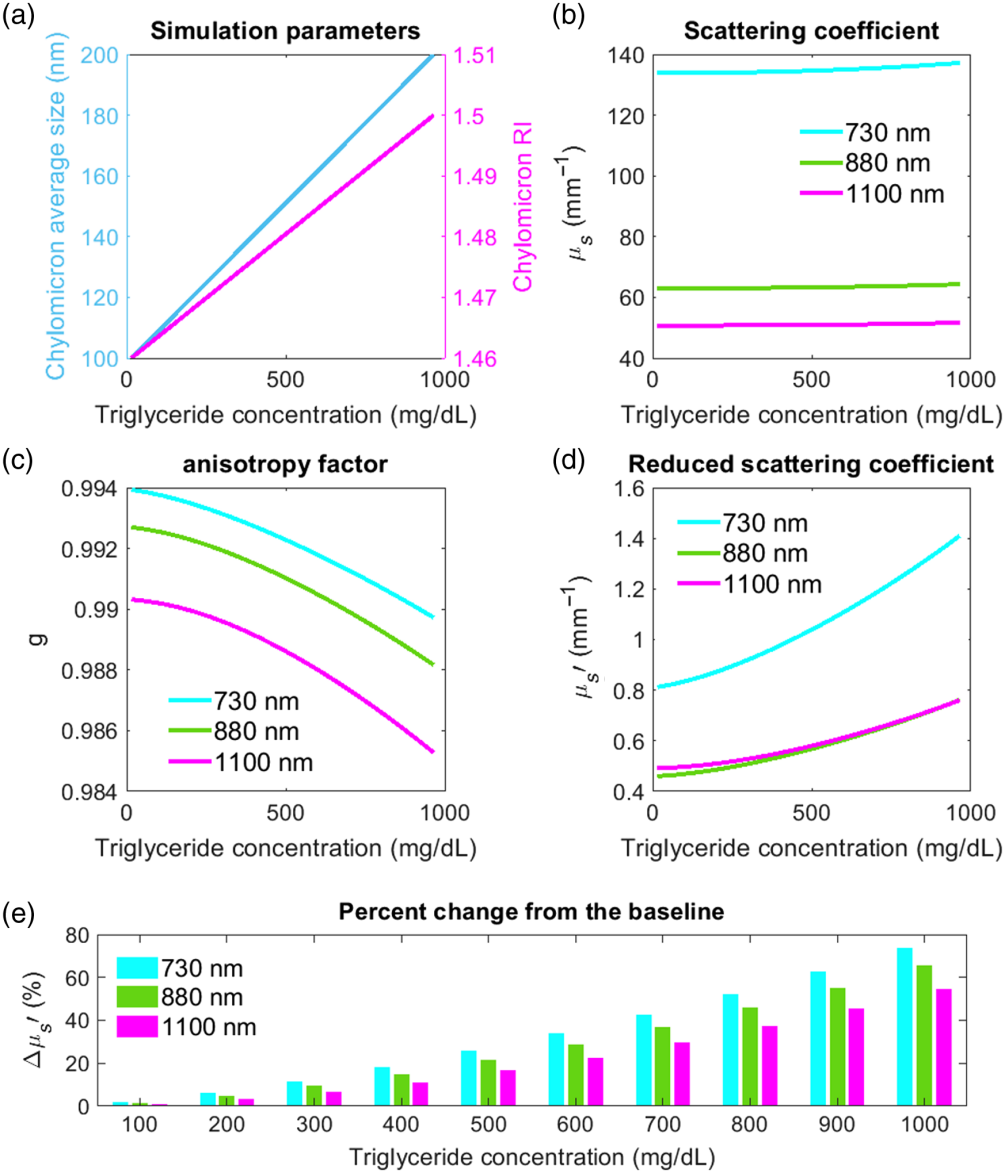
Mie simulation parameters and results for whole blood in fasting and postprandial state. (a) Simulation parameters, (b) blood scattering coefficient ($\mu_{s}$), (c) anisotropy factor, and (d) blood $\mu_{s}^{\prime }$ as the TG content in blood increases. The increase in TG results in higher concentration of chylomicrons, larger chylomicron particles, and higher refractive index. (e) Percent change in blood $\mu_{s}^{\prime }$ from the baseline (first simulation results).

Figures 7(b)–7(d) illustrate the effect of increasing blood TG content on the scattering coefficient ($\mu_{s}$), anisotropy factor (g), and $\mu_{s}^{\prime }$ of blood. The first simulation represents 50  mg/dL of blood TG with an average chylomicron size of 100 nm, and the following 10 simulations show a 50  mg/dL increase in blood TG and a 5 nm increase in average chylomicron size at each step. The refractive index of chylomicron particles increases from 1.46 to 1.5 from the first to the last simulation. The simulation parameters are shown in Fig. 7(a). These simulations suggest that while blood $\mu_{s}$ is not significantly affected by the altered TG content, blood $\mu_{s}^{\prime }$ increases with an increase in blood TG concentration. This increase in blood $\mu_{s}^{\prime }$ is mainly due to the larger scattering angle events as a result of altered TRLs, and hence smaller g parameter compared to the fasting state. Figure 7(e) summarizes the percent change in $\mu_{s}^{\prime }$ for different TG alteration from the baseline simulation.

The effect of lipid alteration on blood $\mu_{s}^{\prime }$ was further simulated by adding the simulated chylomicron and VLDL changes to the experimental measurements of blood $\mu_{s}^{\prime }$. For a healthy subject, we assumed 50  mg/dL of blood TG in the fasting state, which increased by ∼50  mg/dL after a meal. For subjects with type 2 diabetes and hypertriglyceridemia, the baseline blood TG level was assumed to be 200  mg/dL, which increased by 200 and 1000  mg/dL after a meal, respectively. For all subjects, we assumed an average chylomicron size of 100 nm and refractive index of 1.46 at the fasting state, which increased to 200 nm and 1.5 after a meal. Table 3 shows the estimated postprandial $\Delta \mu_{s}^{\prime }$ for healthy subjects, and subjects with type 2 diabetes, and hypertriglyceridemia. Similar to the $\Delta \mu_{s}^{\prime }$ from the whole blood simulation results shown in Fig. 7(d), these simulations also suggest a few percent increase in $\mu_{s}^{\prime }$ for healthy subjects after a meal, and up to a 64% increase in $\mu_{s}^{\prime }$ for subject with hypertriglyceridemia.

**Table 3:** $\Delta \mu_{s}^{\prime }$ from the baseline for different level of ΔTG correspond to different subject population. The Mie simulated $\mu_{s}^{\prime }$ was added to measured bovine blood $\mu_{s}^{\prime }$ at each wavelength.

Subject	ΔTriglyceride	$\Delta \mu_{s}^{\prime }$		
		730 nm	880 nm	1100 nm
Healthy	50 mg/dL	3.85%	3.34%	2.32%
Type 2 diabetes	200 mg/dL	15.23%	13.28%	9.26%
Hypertriglyceridemia	1000 mg/dL	64.37%	55.79%	38.57%

## 7 Discussion

In this work, we investigated the effect of lipoprotein particles on the optical properties of blood. Specifically, we studied chylomicrons and VLDLs, which are the two largest lipoproteins in blood and are highly optically scattering. We used SFDI to experimentally measure the effects of lipoprotein-like particles on the $\mu_{s}^{\prime }$ of blood. Our titration experiment of intralipid in bovine blood showed an increase in $\mu_{s}^{\prime }$ with an increase in intralipid concentration, with larger changes at shorter, NIR wavelengths. The microsphere-in-bovine-blood titration experiment showed an increase in blood $\mu_{s}^{\prime }$ with an increase in particle size, with larger changes for increases in smaller sized beads. The results were then simulated and validated using Mie theory and an empirically derived correction factor for dependent scattering. Next, we generated a distribution for chylomicrons and VLDLs based on prior literature information on their size, refractive index, composition, and concentration. Using this size distribution, we estimated the effects of blood lipid after a meal for different levels of TG alteration, corresponding to healthy, diabetic, and hypertriglyceridemic blood TG content. Our simulations indicate a small increase in blood $\mu_{s}^{\prime }$ (a few percent) in healthy subjects and changes as high as 60% for subjects with hypertriglyceridemia after a meal.

Measurements of blood lipids are important for cardiovascular disease (CVD) assessment. Hyperlipidemia, which refers to the abnormal elevation of blood lipids, including cholesterol and TGs, is one of the major risk factors for CVD and can indicate an increased risk of heart attack, myocardial infraction, and stroke.^2^ Regular screening of blood lipids is essential for early CVD diagnosis, surveillance, and treatment feedback.^30^^,^^31^ Current blood lipid measurements require invasive blood draws, usually after an overnight fast. Recent evidence suggest that postprandial measurement of blood lipids may further improve CVD risk prediction and help to identify pre-diabetic conditions.^32^^,^^33^ Beside the invasive nature of the current technique, blood draws and the following lab-based analysis are resource intensive due to the need of trained technicians and lab equipment. These requirements limit access to blood lipid measurements and prevent those at risk of CVD from undergoing regular blood lipid monitoring.^34^^,^^35^

Measuring the optical properties of blood and their changes over time could offer a potential alternative for assessing blood lipid levels, making regular blood lipid testing more practical and accessible. SFDI has the capability to non-invasively measure blood optical properties through intact skin when combined with multilayer inverse models.^9^ The effect of lipoproteins on optical scattering of blood can potentially provide information about the concentration of the lipoproteins and their response to meals. Additionally, the spectral shape of the scattering coefficient can potentially shed light on lipoprotein sizes and how they change after a meal through Mie analyses.^36^ There may be other invasive and non-invasive techniques that can used to take advantage of the optical scattering changes identified in this work. While longer NIR and SWIR wavelengths are ideal for in vivo measurements due to tissue penetration, shorter wavelengths than those used in this work may be ideal for *ex-vivo* measurements of blood due to the larger expected changes in scattering.

One potential confounder for the use of optical scattering measurements for blood lipoproteins is the impact of hematocrit on blood optical properties. Subjects with different hematocrit levels will have different absorption and scattering properties regardless of their blood lipid status. However, assuming that hematocrit remains constant for a subject after meal, optical measurements of blood can still be used to monitor blood lipid dynamics for each individual. This may require a more advanced modeling to isolate the effect of lipoprotein from other confounding physiological factors, which will be investigated in future studies. Furthermore, the alteration in blood $\mu_{s}^{\prime }$ expected for healthy subjects (a few percent) may be difficult to detect, especially in the *in vivo* state. We also note that our $\mu_{s}^{\prime }$ estimation for different blood TG level could be affected by the simulation parameters and assumptions made here, especially since the literature values for some of these parameters were limited.

In summary, our study investigated the effect of lipoproteins and their alterations on blood optical properties through *ex-vivo* experiments conducted with SFDI and computational modeling using Mie theory. Our findings suggest that lipoproteins are highly optically scattering. Using Mie theory, we were able to estimate the changes in the $\mu_{s}^{\prime }$ of blood following a high-fat meal for individuals with varying medical conditions. The results of this study provide a basis for future investigation of invasive and non-invasive measurements of blood lipoproteins, which may enhance the early detection and management of CVDs.

## Code, Data, and Materials Availability

All relevant code, data, and materials are available from the authors upon reasonable request. Correspondence and requests should be addressed to the corresponding author.
